# Molecular docking based design of Inhibitors for viral Non-Nucleosidase as potential anti-retroviral agents

**DOI:** 10.6026/97320630016736

**Published:** 2020-10-31

**Authors:** Ahmed Alharbi, Khalid Alshaghdali, Amir Saeed

**Affiliations:** 1 Collage of Applied Medical Sciences, Department of Laboratory Sciences, University of Hail, Hail, Kingdom of Saudi Arabia; 2 Department of Medical Microbiology, Faculty of Medical Laboratory Sciences, University of Medical Sciences & Technology, Khartoum, Sudan

**Keywords:** Non-Nucleosidase Inhibitors, Therapeutic agents, Anti-retroviral

## Abstract

Reverse Transcriptase (RT) inhibitors are highly promising agents for use as an effective anti-retroviral therapy (HAART) which is typically a combination of three or four antiretroviral drugs. We used direct drug design approach to discover new chemical entities for the target protein. The validated template of the protein targeting reverse transcriptase PDB ID 1JKH was extracted for three sites hydrophobic, steric, and electronic parameters explain the interactions at the active site by the inhibitors. We used the Zinc library of compounds to explore the possible leads for HAART through RT inhibition. We report 12 new chemical entities with possible activity against the targeted viral protein. These leads will provide new therapeutic means in antiretroviral therapy.

## Background

Reverse Transcriptase (RTase) inhibitors are highly promising agents for use in highly active antiretroviral therapy (HAART) [1] which is typically a combination of three or four antiretroviral drugs [2]. The enzyme Reverse Transcriptase, also known as RNA-dependent DNA polymerase transcribes single-stranded RNA into single-stranded DNA. Unlike other DNA polymerases, RTase is unique as being able to make a complementary DNA strand from an RNA template. These (RTases) are usually found in retroviruses, which use RT to make a DNA copy of their own RNA genome, which is then incorporated into the host cell's genome [3]. The most notorious of these retroviruses is HIV-1, which causes AIDS. The HIV-RTase contains two subunits p66 and p51 with identical amino acid sequences (p66 has 560 amino acid residues and p51 shortened has 440 residues), but are structurally very different [4]. Each of the subunits p66 and p51 have 4 subdomains (fingers, thumb, palm, and connection) which are arranged differently in the two subunits (p66 contains polymerase and RNAse H while p51 lacks RNAse H). The connection and palm subdomains each contain 3 beta-sheets with alpha helices while the thumb subdomains contain 3 alpha-helices. The polymerase active site of subunit p66 is highlighted by 3 catalytic residues (Asp110, Asp185, Asp186) that may play a role in binding metal ions, and hence important for nucleotidyl phosphate interactions [5]. The internal structure or pocket of the RTase enzyme is supposed to be hydrophobically formed by Leu-100, Val-179 and 106, Tyr-181 and 188, Try-229. The classes of compounds that inhibit HIV-RTase are the non-nucleoside reverse transcriptase inhibitors (NNRTIs e.g., Efavirenz, Nevirapine, α-anilinophenylacetamide-APA, Delavirdine) and nucleosidase reverse transcriptase inhibitors (NRTIs e.g. Zidovudine, Didanosine, Zalcitabine). The NNRTI has been shown to bind in a pocket formed between two beta-sheets of the p66 palm, ∼10 Å away from the polymerase active site, and 60 Å from the RNase H region. The NNRTIs although are structurally diverse with relevant binding modes with plasticity in the regions of surrounding proteins to allow some of the unfavorable contacts without changing the binding modes. The active site of HIV-RTase in the bound state with inhibitors (NNRTIs) has volume approximately 600-700 Å with inhibitor occupying ∼250-350 Å [6]. There is a good complementarity of NNI shape to fit in this volume and sometimes it is achieved by conformational rearrangement of the compound. These observations provide an important understanding of the structural basis of the potency of the inhibitors and may suggest possible modifications that could improve interactions with the enzyme. The reverse transcriptase enzyme is the significant target for pharmacophore design as non-nucleoside inhibitor binding pocket (NNIBP) of RTase is known to be flexible and moving to accommodate inhibitors acquiring different shapes depending on the bound inhibitor [7]. Therefore the ligand-based drug design approach where the 3D structural features of ligands are considered to develop pharmacophore which may provide important information for the design of new ligands [8]. In the development of such a pharmacophore, the Non-nucleoside binding site (NNBS) may be considered as a rigid pocket where the 3D arrangement of chemical features in the molecule are considered essential for important binding interactions with the receptor (RTase) [9]. This pharmacophore model may be used for database searching followed by docking experiments to reveal new structural entities as possible inhibitors [10-15]. Ekins et al. explored the 3D-QSAR relationship for inhibition of human ether-a-go-go-related gene for potassium channels [16]. Sechi et al reported novel pharmacophore-based dihydroquinoline-3-carboxylic acids as HIV-1 integrase inhibitors [17]. Dayam et al. reported diketo acid pharmacophore model as integrase inhibitors [18]. M. Y. Li et al. utilized the pharmacophore identification technique for α1A - antagonists [19]. Steindl et al. used pharmacophore modeling in identifying new inhibitors of human rhinovirus coat proteins [20]. Such studies have also been reported for the pharmacophore of the HIV-RT inhibitors e.g. Y. Song et al. reported Common feature pharmacophore for HIV-RT inhibitors along for bisphosphonate inhibitors of ATP-mediated HIV-1 reverse transcriptase [21]. Barrecca et al reported the structure-based pharmacophore identification of new ligands for non-nucleoside reverse transcriptase inhibitors [22]. These studies suggest that the Catalyst generated pharmacophores can be effectively used for rational drug design.

## Materials and Methods:

### Molecular Docking Studies:

Molecular docking was performed using the MolDock module in Molegro Virtual Docker (MVD) software [23] The scoring function of molecular docking in MolDock is based on piecewise linear potentials (PLPs)[24] using protein with PDB ID 1JKH. A re-ranking method was applied to the highest-ranked poses to increase the accuracy of docking. The search algorithm 'MolDock SE' was applied for this analysis, and a population size of 50 and a maximum number of iterations of 1500 were set as parameters. Other parameters were kept as default with the number of runs as 10. Since MVD relies on an evolutionary algorithm, repeated docking runs do not result in precisely the same poses and interactions. To address this intrinsic arbitrariness, ten successive runs were performed and the best three poses were used to visualize further interactions as reported by us previously.

### Database Preparation:

The database of compounds was prepared using the Chemdraw Ultra 12 and minimizing the molecules in sdf format using the online minimization tools for computational studies. The database of 200 compounds was prepared and minimized for the docking experiments along with Molgrow Virtual Docker. The prepared database was added with standard ligands for further verification of the docking protocol.

### Docking Validation:

The validation of the docking was carried out using the co-complexed ligand re-docking to compare the docking poses as well as docking scores. The nevirapine was redocked in the binding site of 1JKH PDB to observe the pose as well as hydrophobic and steric interactions.

## Results and Discussion:

Molecular docking was carried out using the MVD 4.0 software and its standard docking protocol was followed. The binding site was defined 15A around the co-crystal ligand. The amino acids viz. Lys101, Leu100, Thr139, Try383, Val179, Arg172, Glu28 were concentrated as the center of interactions for the database ligands. The internal standard ligands viz. Nevirapine and Efavirenz were analyzed for their interactions as well as their reported pi-pi stackings.

**Figure 1:**
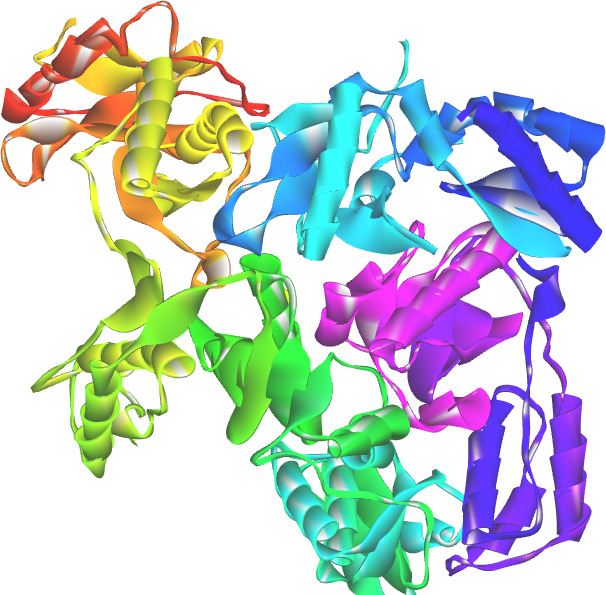
3D structure of target protein HIV-1 reverse transcriptase (PDB: 1JKH)

The database screening of the prepared molecules in .sdf format in the template generated pharmacophore query efavirenz from 1JKH (Figure 1) gives rise to several unknown compounds that have not yet been documented for anti-HIV activity were predicted active. The preliminary filtration by Lipinski's rule of five resulted in the selection of the top 200 hits from Zinc databases. The efavirenz or DMP266 was docked as reference ligand in the binding site of 1JKH using Molegro docking protocols. The Moldock score and rerank scores were employed for analysis of the various scores.

**Figure 2:**
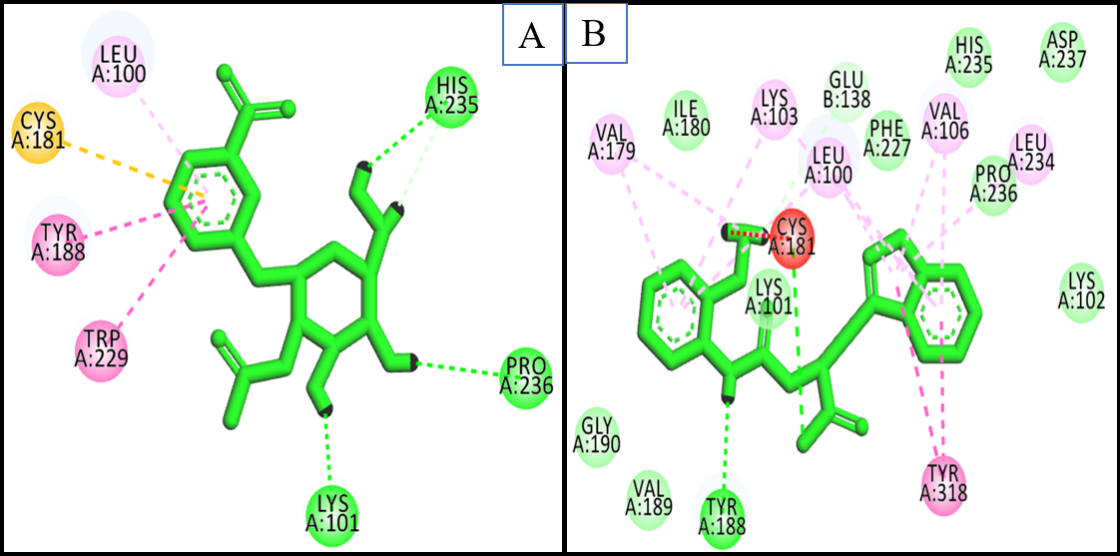
Binding interaction of top compounds with the active site of target protein (1JKH) A) ZINC04042592 B) ZINC02146330

The major interactions of the screened ligands with Lys101, Leu100, Thr139, Try383, Val179, Arg172, Glu28. Similarly, the leads screened from the dataset were docked in the same binding site using both GOLD and Molegro docking protocols. Table 1 (see PDF) enlists the ligands retrieved after the docking along with their GOLD, Moldock scores, binding affinity, and mapping scores with the pharmacophore model respectively. The top 12 leads with higher Gold score and Moldock scores than the reference was identified. All the selected ligands show important binding interactions with the Lys101 and Leu103. The docking analysis clearly shows the important interaction of first-generation NNRTI's with an allosteric hydrophobic pocket (non-nucleoside binding site, NNBS) and bound the enzyme in a "butterfly-like" mode. One wing of this butterfly comprised of electron-rich (phenyl or allyl substituents) moiety and other with interacts through π- π stacking interactions with a hydrophobic pocket formed mainly by the side chains of aromatic amino acids (Tyr181, Tyr188, Phe227, Trp229, and Tyr318). The top screened leads (ZINC02146330 and ZINC04042592) validated by two docking software showed similar interactions with the hydrophobic pocket (Figure 2). The other wing generally heteroaromatic/ aromatic capable of donating or accepting the hydrogen bonds with Lys101 and Lys103. The remaining amino acids as Lys103, Val106, and Val179 give the additional hydrophobicity to the butterfly body.

The core of both structures involves butterfly conformation in the binding site and the hydrophobic and electronegative interactions due to cyclopropyl ring are supported well by phenyl ring and carboxylic acid substitution. The better scores in terms of docking for these ligands were due to additional interactions of leads, which tend to stabilize binding addition to important core interactions. Thus, these potential leads comprising important pharmacophore features required for selective reverse transcriptase inhibition. The comparable docking figures showed the interactions of nevirapine and top screened lead with important interactions and the respective binding scores in terms of Gold score, Moldock score, and Rerank scores are presented in Table 1 (see PDF).

## Conclusion

In this study, we report 12 new chemical entities with possible activity against the targeted viral protein for further consideration. These leads will provide new therapeutic means for antiretroviral therapy.
